# Novel Insights into Regulation of Human Teeth Biomineralization: Deciphering the Role of Post-Translational Modifications in a Tooth Protein Extract

**DOI:** 10.3390/ijms20164035

**Published:** 2019-08-19

**Authors:** Vaibhav Sharma, Komal Rani, Ajoy Roychoudhury, Amita Chawla, Fredrik Nikolajeff, Saroj Kumar

**Affiliations:** 1 Department of Biophysics, All India Institute of Medical Sciences (AIIMS), New Delhi 110029, India; 2 Department of Oral and Maxillofacial Surgery, Center for Dental Education and Research (CDER), All India Institute of Medical Sciences (AIIMS), New Delhi 110029, India; 3 Department of Engineering Sciences, Uppsala University, 75105 Uppsala, Sweden

**Keywords:** human teeth, biomineralization, intrinsically disordered proteins, post-translational modifications, scanning electron microscopy, transmission electron microscopy

## Abstract

The importance of whole protein extracts from different types of human teeth in modulating the process of teeth biomineralization is reported. There are two crucial features in protein molecules that result in efficient teeth biomineralization. Firstly, the unique secondary structure characteristics within these proteins i.e. the exclusive presence of a large amount of intrinsic disorder and secondly, the presence of post-translational modifications (PTM) like phosphorylation and glycosylation within these protein molecules. The present study accesses the structural implications of PTMs in the tooth proteins through scanning electron microscopy and transmission electron microscopy. The deglycosylated/dephosphorylated protein extracts failed to form higher-order mineralization assemblies. Furthermore, through nanoparticle tracking analysis (NTA) we have shown that dephosphorylation and deglycosylation significantly impact the biomineralization abilities of the protein extract and resulted in smaller sized clusters. Hence, we propose these post-translational modifications are indispensable for the process of teeth biomineralization. In addition to basic science, this study would be worth consideration while designing of biomimetics architecture for an efficient peptide-based teeth remineralization strategy.

## 1. Introduction

Protein molecules play a crucial role by working downstream to genes in every living system. They carry out diverse functions ranging from transmitting signal molecules to building muscles. One such seemingly interesting process, modulated by proteins, is biomineralization. Biomineralization is a process by which inorganic ions are accumulated in a systematic way on organic templates [1]. Biomineralization is still considered as a scientific puzzle. Representative examples include systems like bone and teeth formation. An important group of proteins involved in biomineralization is inherent/intrinsically disordered proteins (IDPs) [2,3]. They are proteins that in the native state are partially or completely devoid of a strictly defined tertiary structure. IDPs occur as a heterogeneous population molecule with high conformational dynamics. High accidental load, modifiability susceptibility cations, flexibility and their ability to interact with many different partners make these proteins especially adapted to perform diverse functions in the process of biomineralization. Although proteins play such an important role in the biomineralization, their mechanism of action is still not well explained. Some earlier reports suggest that IDPs undergoes various conformational transitions upon binding with calcium ions [4,5] and a variety of other substrates [6]. Many proteins involved in biomineralization are modified at the post-translational level, which has a significant impact on their biomineralizing activity [7,8,9]. In fact, post-translational modifications (PTMs) were shown to have a fondness for disordered regions in many proteins with known nuclear magnetic resonance (NMR) structures [10]. Modification like phosphorylation and glycosylation are reported to affect the mineralization ability of individual proteins [11,12]. The post-translationally modified structures will fold differently when they bind to their partners. The importance of PTMs for mineralization was shown since the non-phosphorylated forms did not interact with hydroxyapatite (HA) or Ca^2+^ ions [8,13].

To better understand the effects of PTMs like phosphorylation and glycosylation, this study addresses their effect on the biomineralization capacity of the isolated tooth protein extract.

Although a great deal of experimental efforts has been focused on the role of amelogenin in affecting the growth of calcium phosphate crystals [14,15,16], there have only been a few studies to examine the effect of whole protein extract from teeth, which imitates the *in vivo* process of teeth biomineralization. Our recent report highlighted the importance of whole protein extracts from different types of human teeth in modulating the process of teeth biomineralization [17]. We established the presence and importance of intrinsically disordered proteins (IDPs) within different protein extracts from healthy human teeth. The present study was carried out using whole protein extract from human teeth, their dephosphorylated and deglycosylated counterparts were also used to check for their capacity to functionally modulate teeth biomineralization. In the current study, we report that PTMs like phosphorylation and glycosylation in the proteins of human teeth are very much indispensable for systematic biomineralization of teeth.

## 2. Results and Discussion

The protein extract from human teeth was successfully isolated and purified as previously described [17]. Before the *in vitro* mineralization assay setup, the protein extract was checked by running onto a 12% Sodium Dodecyl Sulfate Polyacrylamide gel electrophoresis (SDS-PAGE), followed by the standard silver staining of proteins. The zeta potential measurements were also done to measure the overall surface charge of the protein molecules (Supplementary Figure S1). Normal tooth protein (NM) showed the average potential of −16.5 mV whereas in the case of dephosphorylated protein (DP), the potential decreased to −11.2 mV. This resulted from the removal of some phosphate groups. In the case of deglycosylated protein (DG), the zeta potential was −18.5 mV, this increase in potential could be attributed to the successful removal of glycosylated groups of proteins.

### 2.1. Scanning and Transmission Electron Microscopy (SEM and TEM) Analysis

**Figure 1:**
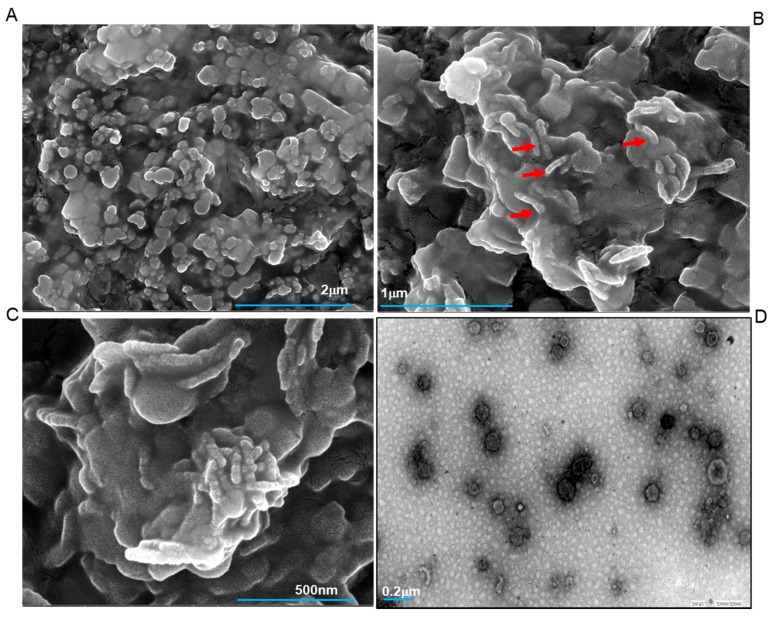
FESEM micrograph of calcium phosphate mineral products in the presence of normal tooth protein extract (**A**–**C**). The red arrows shown in (**B**) corresponds to an elongated structure of calcium phosphate. The HRTEM image of the biomineralization process is shown in (**D**). All scale bars with size are indicated at the lower part of images.

*In vitro* mineralization assay was performed as previously reported [17,18]. Normal protein extract, dephosphorylated extract, and deglycosylated extract were taken in addition to calcium phosphate. Physiological Ca/P molar ratio of 1.66 was taken. The reaction setup was kept at 37 °C for 6 h. The samples were then taken on the carbon-coated grids after 6 h of *in vitro* mineralization setup. Figure 1A–C shows the High-Resolution Field Emission Scanning Electron Microscopy (HR FESEM) images at various magnifications with scale bars. In Figure 1A–C, the process of mineralization can be clearly seen. The elongated structures shown with red arrows depict calcium phosphate crystals which are seen to interact extensively with the protein molecules (round particles) to give rise to higher-order assemblies. Figure 1A shows the surface view of the mineral formation at a magnification of 50,000×. Here, we can clearly observe the small assemblies interacting with each other. In Figure 1B, the interactions between calcium phosphates and proteins can be clearly visualized. At a magnification of 200,000×, Figure 1C depicts a detailed view of the complicated process of mineralization, the FESEM captured the ongoing mineralization process involving a complex interaction of organic molecules and their inorganic counterparts.

Several reports on enamel protein amelogenin show similar results on the regulation of calcium phosphate *in vitro* [14,15,16]. This is the first report on the whole protein extracts isolated from human teeth, which in our view, should more closely imitate the *in vivo* process of mineralization. Figure 1D is a High-Resolution Transmission Electron Microscopy (HR TEM) image of the sample, this shows the formation of round assemblies in the presence of calcium phosphates, some of these are interacting themselves.

**Figure 2:**
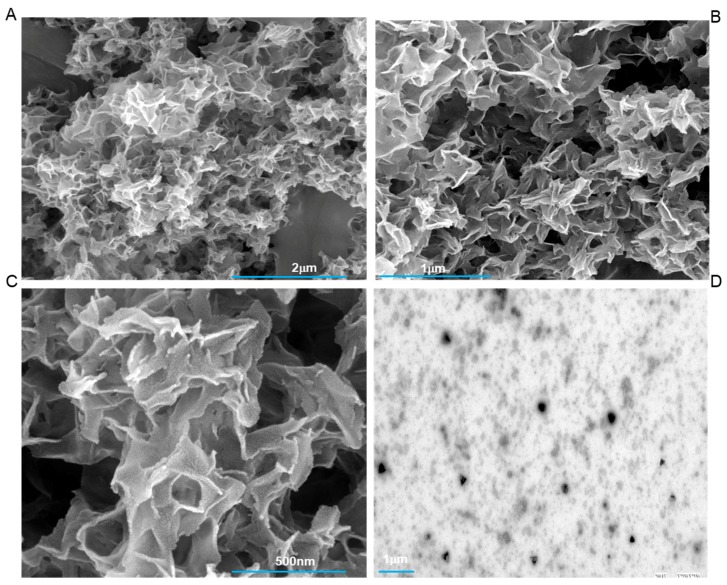
FESEM micrograph of calcium phosphate mineral products in the presence of dephosphorylated tooth protein extract. (**A**–**C**) shows the absence of organized mineralization and results in only random assemblies. The corresponding TEM image with the dephosphorylated extract is shown in (**D**). All scale bars with size are placed at the lower part of images.

Figure 2A–D shows the results with the dephosphorylated extract of tooth proteins. There is a clear absence of compact mineralization assemblies within these extracts. Even at higher magnifications (200,000×) (Figure 2C), we could not observe any significant association of protein extract with the inorganic counterparts. In comparison of these results with Figure 1A–D, we can conclude that phosphorylation has a significant effect in regulating the process of teeth mineralization. These results do echo with several other studies done on individual protein like amelogenin [13], and dentine matrix protein 1 [8], from enamel and dentine respectively, which have shown that even a change in the single phosphorylation site has an enormous effect on the ability to form/modulate higher-order mineralization assemblies.

**Figure 3:**
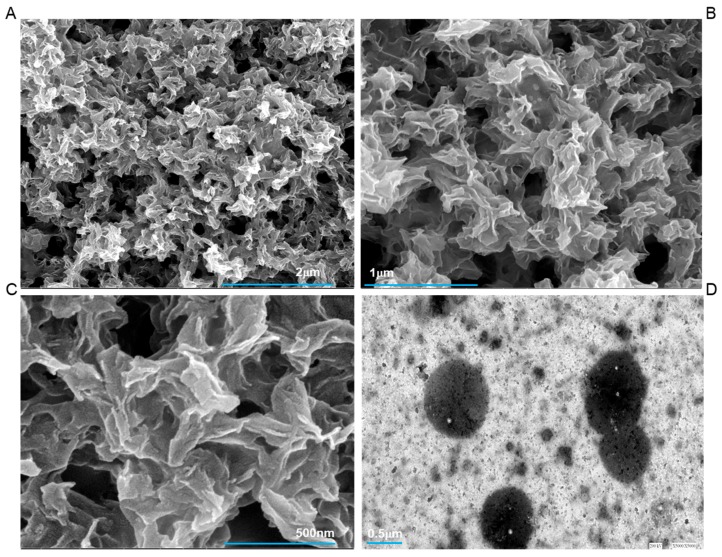
FESEM micrograph of calcium phosphate mineral products in the presence of deglycosylated tooth protein extract. (**A**–**C**) shows the absence of systematized mineralization and results in only random aggregates of calcium and phosphates. The corresponding TEM image with the deglycosylated extract is shown in (**D**). All scale bars with size are placed at the lower part of images.

Figure 3A–D shows the results with the deglycosylated extracts of tooth proteins. Under these conditions, SEM and TEM images show that this treated extract lacks the capacity to regulate ordered mineralization and have only resulted in uncontrolled, random depositions. We have not visualized the interconnected structures of protein and calcium phosphate, as observed in the case of Figure 1A–C. Although a lot of work has been done to see the effect of dephosphorylated protein on their respective mineralization capacity, studies revealing the effect of glycosylation are insufficient. Though the presence of glycosylated proteins has been well established only some studies have reported their role in enamel mineralization [19] and these were not microscopy based.

**Figure 4:**
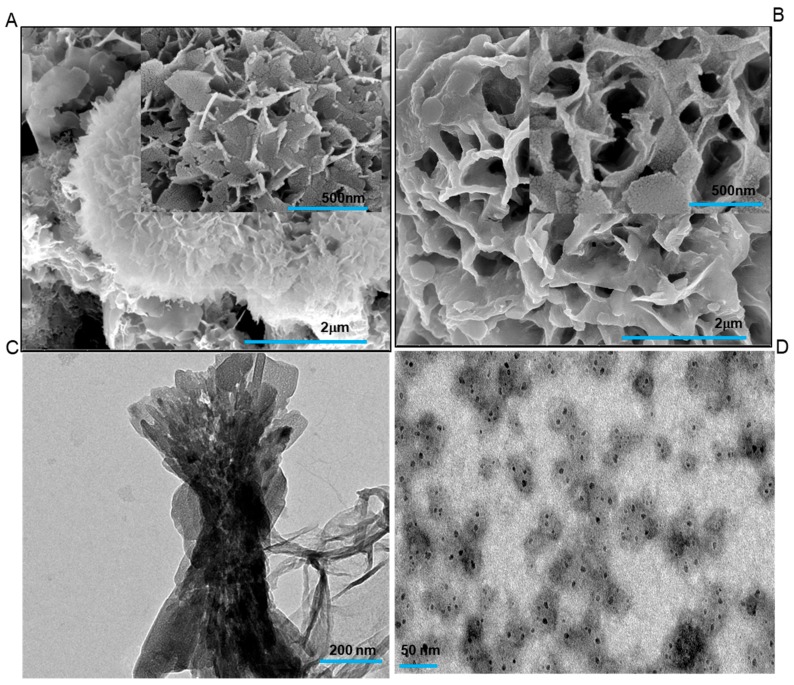
(**A**) Control FESEM micrograph of calcium phosphate mineralization in the absence of any protein. The inset shows the calcium phosphate flakes at 500 nm, whereas the representative TEM image of calcium phosphate control is shown in (**C**). FESEM micrograph at (**B**) shows the absence of mineralization in the presence of Bovine serum albumin (BSA) protein and calcium phosphates. (**D**) shows the TEM image in the presence of BSA and calcium phosphates.

Figure 4 panel shows the control TEM and SEM images with only calcium phosphate and BSA (control protein). Figure 4A shows the typical calcium phosphate flakes formation and the inset shows a corresponding image at 500 nm scale bar. Figure 4C depicts the TEM image of the calcium phosphate control reaction. Figure 4B and Figure 4D shows the mineralization reaction with the BSA as a control protein. BSA was taken as a protein control, to establish the specificity of the tooth proteins towards calcium phosphates. The negative result in BSA associated calcium phosphate reaction proves the role of tooth proteins in the mineralization process. In all the controls, the mineralization pattern was different from the ones observed in the presence of tooth proteins. BSA control images proved the specificity of tooth protein extracts towards the process of biomineralization. We have also put only protein controls without calcium phosphate during *in vitro* mineralization assays for 6 h, to rule out the aggregation of the protein. The SEM and TEM images showed no aggregation of protein molecules (Supplementary Figure S2).

### 2.2. Nanoparticle Tracking Analysis (NTA)

**Figure 5:**
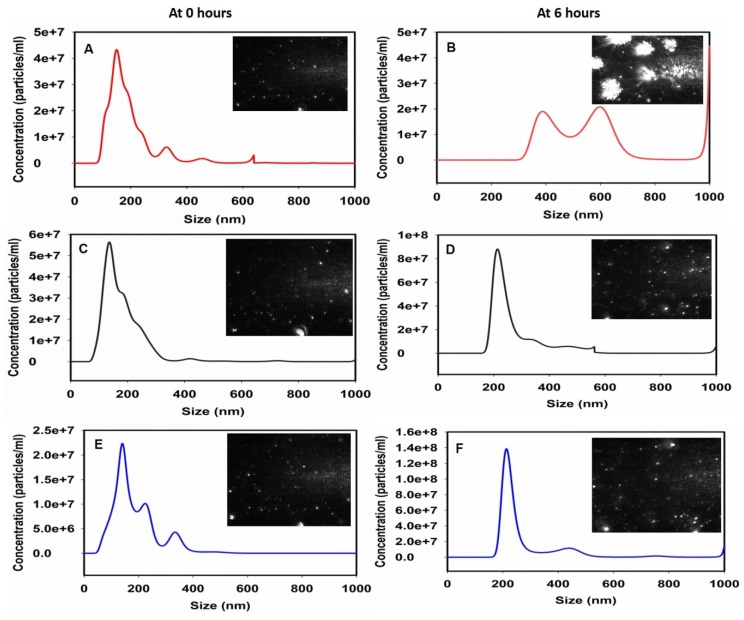
Nanoparticle tracking analysis (NTA) size measurement experiments to observe the biomineralization. (**A**) Tooth protein extract at 0 h. (**B**) Tooth protein extract in the presence of calcium phosphate at 6 h. (**C**) Dephosphorylated tooth protein extract at 0 h and (**D**) depicts the 6 h image in the presence of calcium phosphate. (**E**) Deglycosylated tooth protein extract at 0 h and the corresponding mineralization effect after 6 h is seen at (**F**). All the figures have inset showing representative images taken by NTA attached camera at 0- or 6-h time points.

Nanoparticle tracking analysis was done to check the size measurement of the formed assemblies. The size was measured at 0 h and after 6 h of *in vitro* mineralization setup. Figure 5A,B show the effect of tooth proteins in the regulation of calcium phosphate mineralization at 0 h and 6 h respectively. The increased size after 6 h clearly denotes the efficient mineralization capabilities of the native tooth protein extract. These data were consistent with our previous findings [17]. Figure 5C,D show the result of dephosphorylation at 0 and 6 h respectively. There is no significant increase in the size of the particles which indicates a lack of proper mineralization through these extracts. Similar results were observed in the presence of deglycosylated protein extract at above time points (Figure 5E,F). Overall, NTA results were well correlated with the electron microscopy results (Figure 1) and marked the importance of PTMs within these tooth protein extracts to carry out the process of teeth biomineralization.

## 3. Materials and Methods

### 3.1. Isolation of Proteins from Human Teeth

This study was approved by the Institute Ethics Committee for Post Graduate Research of All India Institute of Medical Sciences (Ref no. IECPG-387/29.06.2016, RT-02/27.06.19). The study was conducted in accordance with the Declaration of Helsinki. Written informed consent was received from all participants. Human teeth samples used in this study were from living, healthy individuals, which were extracted due to orthodontic reasons. The age group was 18–40 years. Samples were collected from the Center for Dental Education and Research (CDER), AIIMS, New Delhi. Collected teeth were kept at 10% NaCl to inhibit bacterial growth and contamination. The isolation of protein extracts from human teeth was done as previously reported [17]. Briefly, teeth were washed thoroughly and were crushed in a pestle and mortar in the presence of liquid nitrogen until a fine powder was formed. 10 g of powder was taken and mixed with 20 mL of demineralization solution containing 0.1 M EDTA, 100 mM NaCl and protease inhibitors (Roche, Penzberg, Germany) at 20 °C for 2 days, centrifuged at 10,000 rpm and collected supernatant was dialyzed against HEPES buffer, pH 7.4. The clarified supernatant was further concentrated by the membrane-based cut off filters (3.5 kDa). The protein concentration was measured by BCA kit (Pierce, IL, USA) according to the manufacturer’s instructions. The sample was run on 12% SDS-PAGE and afterward stained using silver nitrate staining procedure.

### 3.2. Dephosphorylation of the Tooth Protein Extract

Dephosphorylation of protein extract was done by using bovine intestinal alkaline phosphatase (Cat.no.P0114, Sigma Aldrich, St. Louis, MO, USA). 100 units of alkaline phosphatase were incubated with 400 μg of protein extract in 5 mM Tris pH 7.9, 10 mM NaCl, 1 mM MgCl_2_, and 0.1 M dithiothreitol (DTT) for 30 min at 30 °C. The reaction was stopped by addition 5 × sample buffer and boiling prior to SDS-PAGE analysis.

### 3.3. Deglycosylation of the Tooth Protein Extract

Deglycosylation of protein extract was done by using Protein Deglycosylation Mix II (NEB). Protein Deglycosylation Mix II (P6044) was procured from New England Biosciences (NEB, Massachusetts, USA). Deglycosylation setup was done as per manufacturer’s suggested protocol. Briefly, 100 μg of protein extract was dissolved in 40 μL distilled water. Five microliters of 10X Deglycosylation Mix Buffer 1 was added. Then, 5 μL of Protein Deglycosylation Mix II was added. The solution was mixed gently. The reaction mixture was incubated at 25 °C for 30 min and then it was transferred to 37 °C for 16 h.

### 3.4. Zeta Potential Measurements

The electrokinetic potential of protein solution, commonly known as Zeta potential was measured using the Zetasizer Nano Series (Malvern Instruments, Worcestershire, UK). A similar dilution of all protein solutions in distilled water was added to a specialized capillary cell for measurement. Zeta potential measurements reported are a mean ± standard error of three repeat experiments.

### 3.5. In Vitro Mineralization Assay

Stock solutions of calcium (50 mM) and phosphate (5 mM) were prepared using CaCl2 (Sigma, St. Louis, MO, USA, >99.0% pure) and KH2PO4 (Sigma, >99.0% pure). All solutions (except protein extracts) were filtered (0.22-μM filters, Millipore, Darmstadt, Germany) before use. The KH_2_PO_4_ solution was adjusted to pH 7.5–11.2 at 25 °C, using a small volume of KOH. The precise pH value was selected by design so that the reaction solution would have an initial pH~7.4 at 37 °C upon mixing all solution components. Aliquots of calcium and pH-adjusted phosphate solution were sequentially added to protein solutions to yield final concentrations of 2.5 mM Ca^2+^, 1.5 mM Pi, and 0.2–2.0 mg/mL protein, with a final volume of 100 μL, as previously reported [15]. Samples were then placed in a thermostated water bath adjusted to 37 °C. Initial pH values were set to ~ pH 7.4. To minimize evaporation, the reaction tube was tightly sealed with a cap or parafilm. Each experiment was carried out using two identically prepared samples. One sample was visualized in the light microscope(Nikon SMZ 1500, New York, NY, USA) attached to polarization accessory; another sample proceeded for Nanoparticle size-based analysis.

### 3.6. High-Resolution Field Emission Scanning Electron Microscopy (HR FESEM)

HR FESEM was performed on NOVA NANOSEM-450, FEI (Hillsboro, OR, USA) with Zeiss lens. Aliquots from 96 well plate kept for mineralization in the presence of teeth protein extract were taken. Five microliters of each sample type were pipetted onto carbon-coated grids (Agar Scientific, Essex, UK). The grids were air-dried for several minutes. HR FESEM was operated at high voltage (HV) of 10.00 kV. Images obtained were from 100 nm to 10 μM and magnification ranging from 10,000× to 400,000×. Energy-dispersive X-ray (EDS or EDX) was also performed with the attached accessory within the same instrument. For EDS, HV of 20 kV was used.

### 3.7. High-Resolution Transmission Electron Microscopy (HRTEM)

Aliquots from 96 well plate kept for mineralization in the presence of tooth protein extract were taken. Each sample was analyzed on TECHNAI G2, S-Twin HRTEM (Brno, Czech Republic), FEI company. Five microliters of each sample were placed on a carbon-coated grid (Agar Scientific, Essex, UK). Air drying was done for a few minutes. HRTEM was operated at 100 kV, in bright field.

### 3.8. Nanoparticle Tracking Analysis (NTA)

NTA measurements were performed with a NanoSight LM20 (NanoSight, Amesbury, UK), equipped with a sample chamber with a 640-nm laser. The samples were injected into the sample chamber with sterile syringes (BD Discardit II, NJ, USA) until the liquid reached the tip of the nozzle. All measurements were performed at room temperature. The software used for capturing and analyzing the data was the NTA 2.0 Build 127. The values obtained were that of concentration (particles/mL) and size. These were plotted in SigmaPlot 12.0 software.

## 4. Conclusions

In summary, the present study shows that posttranslational modifications like phosphorylation and glycosylation in tooth proteins may have a potentially important function in the regulation of teeth biomineralization and formation of higher-order assemblies. The phosphorylation status of some individual tooth proteins is well known to modulate the process of mineralization. In these cases, the change to a more ordered structure after phosphorylation, interactions with either calcium ions or hydroxyapatite was verified by various biophysical techniques. These changes in conformation accompanying with posttranslational modifications could facilitate nucleation and further growth of mineral crystals. In line with earlier studies, we report the effects of dephosphorylation of whole protein extracts from human teeth, such an extract should imitate the actual in vivo mineralization process more closely.

Although the presence of glycosylated proteins is established within human teeth, we could not find any concrete literature describing their role in mineralization. Based on the noted differences in effects on mineralization, we propose for the first time that the glycosylation is also critical for efficient mineralization of teeth.

## Supplementary Materials

Supplementary materials can be found at https://www.mdpi.com/1422-0067/20/16/4035/s1.
