# Supplementary material for: Novel Insights into Regulation of Human Teeth Biomineralization: Deciphering the Role of Post-Translational Modifications in a Tooth Protein Extract
Source: Int J Mol Sci. 2019 Aug 19;20(16):4035. doi: 10.3390/ijms20164035 (PMC6720696; doi:10.3390/ijms20164035)
Supplement: Supplementary file 1 [file ijms-20-04035-s001.pdf]

Supplementary Materials: Supplementary materials can be found at www.mdpi.com/xxx/s1.

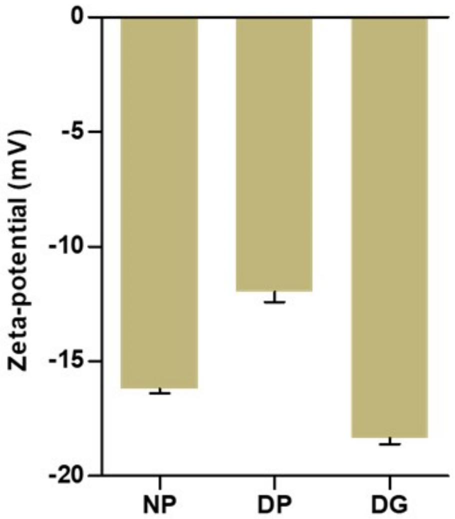

Figure S1. Zeta potential measurements of normal tooth protein (NP), dephosphorylated protein (DP) and deglycosylated protein (DG). The data represent the mean ± standard error of three repeats.

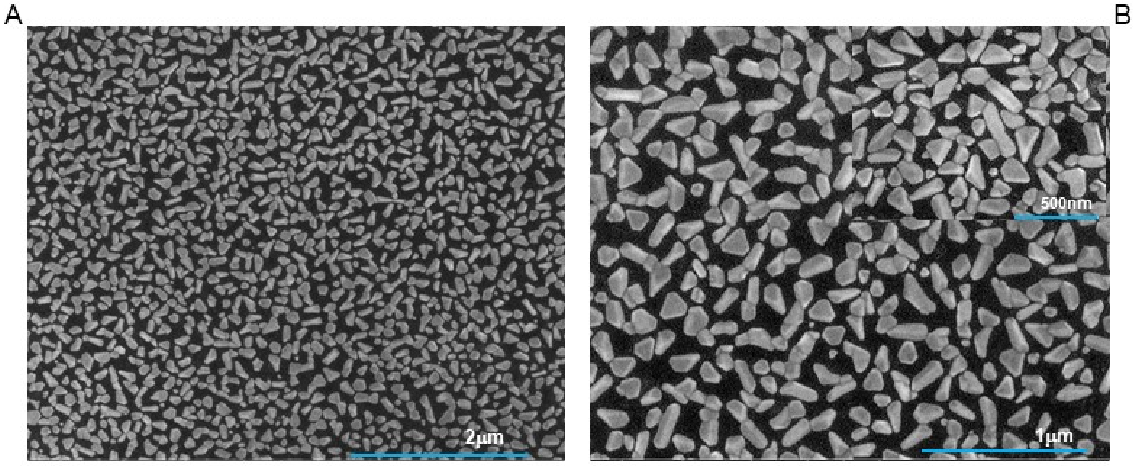

Figure 2. FESEM micrographs of protein only control studies (absence of calcium phosphate). **A** shows protein molecules at 2 µM scale. **B** shows the enlarged version at 1 µM. The inset in **B** shows the details at 500 nm.
